# Pacemaker Placement in Persistent Left Superior Vena Cava

**DOI:** 10.7759/cureus.1311

**Published:** 2017-06-05

**Authors:** Murtaza Sundhu, Mubbasher Syed, Sajjad Gul, Bilal Saqi, Robert Mosteller

**Affiliations:** 1 Internal Medicine Residency, Fairview Hospital, Cleveland Clinic, USA; 2 Electrophysiology, Fairview Hospital, Cleveland Clinic, USA

**Keywords:** pacemaker, electrophysiology, left superior vena cava

## Abstract

Persistent left superior vena cava (PLSVC) is a rare disorder which is asymptomatic and hence is usually discovered while performing interventions through the left subclavian vein. We present a case of a 78-year-old male who was undergoing elective placement of a permanent pacemaker for tachycardia – bradycardia syndrome with post-conversion pauses of up to nine seconds. After achieving access through the left subclavian vein the wire kept on going on the left side of the chest instead of crossing the midline to the right side. The wire was removed and contrast venography was done, PLSVC with dilated coronary sinus emptying into the right atrium was confirmed. There was some difficulty in passing the lead to the right ventricle even with the acute curve in the stylet. The sheath size was increased and a longer deflectable sheath was used and with the tip of the lead anteriorly the right ventricle was cannulated and the lead was affixed. There were good sensing and pacing parameters. Post procedure chest x-ray was done and the patient was discharged without any complications.

## Introduction

Persistent left superior vena cava (PLSVC) is a rare disorder which is present in 0.1–0.5% of the general population [1-3]. It is the most common disorder of the systemic venous system. PLSVC results when the left anterior cardinal vein fails to obliterate [4]. PLSVC is most frequently found in association with other congenital heart diseases and the prevalence goes up to 12.9% [5]. Atrial septal defect, ventricular septal defect, Tetralogy of Fallot, coarctation of aorta and transposition of great arteries are the most common congenital heart diseases associated with PLSVC. VACTERL and CHARGE syndromes are the most common multi-organ congenital disorder associations [6].

## Case presentation

We present a case of a 78-year-old male undergoing elective permanent pacemaker placement for tachycardia-bradycardia syndrome. He had a history of atrial fibrillation (AF) for which he had undergone five synchronized cardioversions over the previous two years, atrial flutter for which he had undergone cavotricuspid isthmus ablation two years earlier, and a remote resolved tachycardia-induced cardiomyopathy. He also had hypertension and obstructive sleep apnea, but no known coronary artery disease or stroke. He was chronically anticoagulated with warfarin and on dofetilide for suppression of AF, and also had significant sinus bradycardia and some documented post-conversion pauses of up to nine seconds, necessitating pacemaker placement.

After achieving access through the left subclavian vein and placement of a Seven French (7 Fr) sheath into the vessel, a 58-cm active fixation pacemaker lead was introduced. Rather than crossing the midline, it repeatedly coursed caudally on the left side of the mediastinum, raising the suspicion of a PLSVC. The lead was removed, and contrast injected, with venography confirming drainage into a markedly dilated coronary sinus before emptying into the right atrium (Video 1).

**Video 1:** Venography

**Figure 1:**
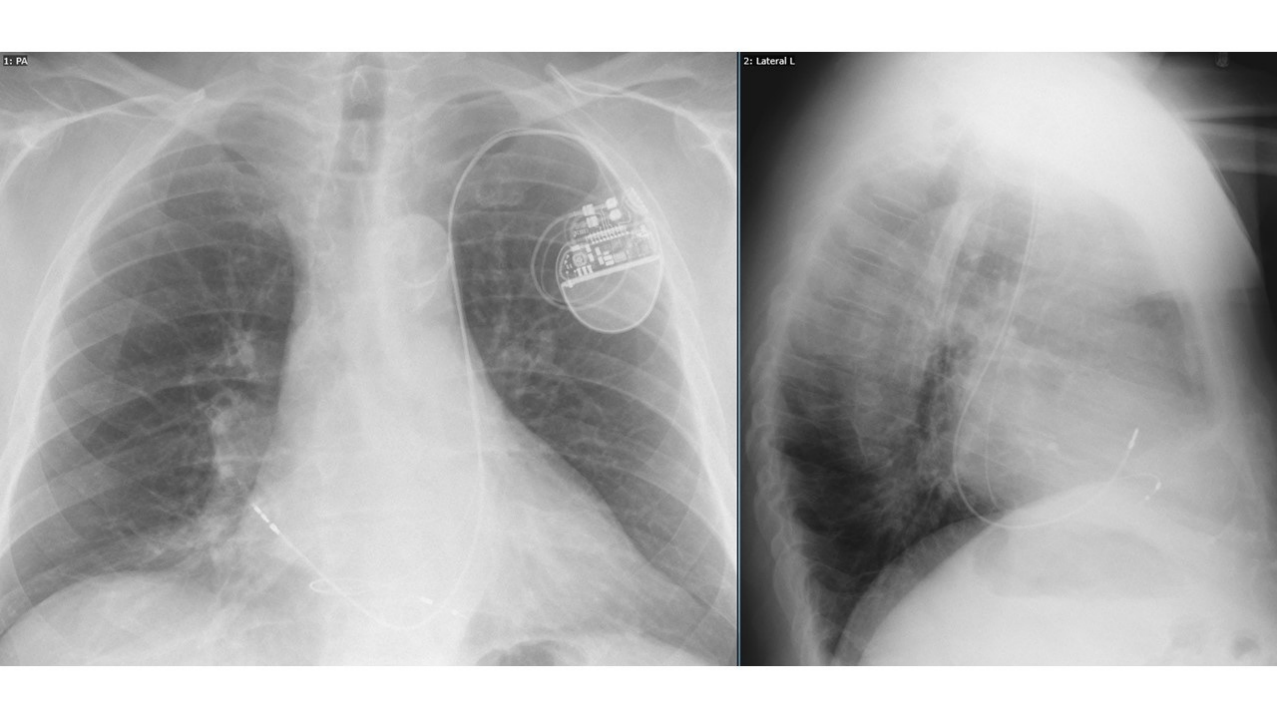
Post-procedure chest x-ray

The 58-cm lead was then passed through this venous system into the right atrium (RA), but it could not be easily manipulated across the tricuspid valve into the right ventricle (RV), even with an acute curve in the stylet. The lead was removed, and the 7 Fr sheath replaced by a long 8.5 Fr deflectable sheath which was advanced through the coronary sinus ostium into the RA, and the tip directed anteriorly. The 58-cm lead was then successfully passed into the RV and affixed to the ventricular wall, after which the long sheath was slit and removed. A 52-cm active fixation atrial lead was then passed through a separate 7 Fr left subclavian sheath through the large coronary sinus and directly to the mid-lateral wall of the RA, where it was affixed. Both atrial and ventricular sensing and pacing parameters were excellent. The leads were attached to a pacemaker generator. A post-procedure fluoroscopy is shown in Video 2 and chest radiograph is shown in Figure 1. Following successful pacemaker placement, the patient’s beta blocker therapy was resumed.

**Video 2:** Fluoroscopic view after the procedure

## Discussion

PLSVC is usually asymptomatic and is often an incidental finding during peripherally-inserted central catheter (PICC) line or pacemaker placement. PLSVC is also associated with arrhythmias and can be a cause of atrial fibrillation [7]. Many procedural encounters with this unforeseen anomaly have been reported in the literature, and may increase in prevalence with an increase in left-sided transvenous procedures. PLSVC can be diagnosed by transthoracic echocardiography, transesophageal echocardiography, contrast venography, computed tomography venography and magnetic resonance venography, but these are not routine prerequisites to pacemaker placement or PICC line insertion.

For pacemaker placement, the greatest challenge is crossing the tricuspid valve to place the ventricular lead [8], which may rarely be complicated by dissection or perforation of the thin-walled coronary sinus. In one case report, tricuspid valve was crossed by making a loop of the ventricular lead against the lateral wall of the right atrium and the stylet was made semicircular in shape to facilitate the passage into the right ventricle [9]. PLSVC co-exists with right-sided superior vena cava in 80–90% of the cases [1]. If lead placement cannot be performed satisfactorily, a right subclavian venous approach may be necessary for procedural success via the right superior vena cava.

We used a long 8.5 Fr deflectable sheath rather than the 7 Fr sheath and the tip of the lead was placed anteriorly to cross the tricuspid valve and was successful.

## Conclusions

A PLSVC is an uncommon and asymptomatic congenital anomaly which is occasionally and unexpectedly encountered during routine pacemaker placement. The anatomic challenges presented by this variant can generally be overcome by an experienced operator who carefully manipulates the leads through the coronary sinus and into appropriate anatomic positions using long guide sheaths and/or customized stylet shapes.
